# The Procedural and Clinical Outcomes of Rotational Atherectomy in Patients Presenting With Acute Myocardial Infarction

**DOI:** 10.3389/fcvm.2022.846564

**Published:** 2022-03-18

**Authors:** Yu-Wei Chen, Chih-Hung Lai, Chieh-Shou Su, Wei-Chun Chang, Chi-Yen Wang, Wei-Jhong Chen, Tzu-Hsiang Lin, Kae-Woei Liang, Tsun-Jui Liu, Wen-Lieng Lee

**Affiliations:** ^1^Cardiovascular Center, Taichung Veterans General Hospital, Taichung, Taiwan; ^2^Institute of Clinical Medicine, National Yang Ming Chiao Tung University, Taipei, Taiwan; ^3^Department of Cardiology, Feng Yuan Hospital, Ministry of Health and Welfare, Taichung, Taiwan; ^4^Department of Life Science, Tunghai University, Taichung, Taiwan; ^5^Institute of Medicine, Chung Shan Medical University, Taichung, Taiwan; ^6^Department of Cardiology, Taipei Veterans General Hospital Hsinchu Branch, Hsinchu, Taiwan; ^7^Department of Medicine, National Yang-Ming University School of Medicine, Taipei, Taiwan

**Keywords:** percutaneous coronary intervention, rotational atherectomy, acute coronary syndrome, acute myocardial infarction, coronary artery disease

## Abstract

**Background:**

Rotational atherectomy (RA) is an indispensable tool used for calcified lesion preparation in percutaneous coronary intervention (PCI). However, use of RA in the setting of acute myocardial infarction (AMI) is challenged with limited clinical data.

**Objectives:**

This study aims to retrospectively investigate the procedural results, periprocedural complications, and clinical outcomes of RA in patients with AMI.

**Methods:**

All possible consecutive patients who received RA in AMI from January 2009 to March 2018 in a single tertiary center were analyzed retrospectively. Patients without AMI during the study period were also enrolled for comparison.

**Results:**

A total of 121 patients with AMI (76.0 ± 10.8 years, 63.6% males) and 290 patients without AMI were recruited. Among the AMI group, 81% of patients had non-ST-elevation myocardial infarction (NSTEMI) and 14% presented with cardiogenic shock. RA could be completed in 98.8% of patients in the AMI group and 98.3% in the non-AMI group (*p* = 1.00). The periprocedural complication rates were comparable between the AMI and non-AMI groups. The risks of in-hospital, 30-day, 90-day, and 1-year cardiovascular major adverse cardiac events (CV MACE) were significantly higher in the AMI group compared with the non-AMI group (in-hospital 13.2 vs. 2.8%, *p* < 0.001; 30-day 14.2 vs. 4.5%, *p* < 0.001; 90-day 20.8 vs. 6.9%, *p* < 0.001; 1-year 30.8 vs. 19.1%, *p* = 0.01). AMI at initial presentation and cardiogenic shock were predictors for both in-hospital CV MACE and 1-year CV MACE in multivariable binary logistic regression analysis. Other predictors for 1-year CV MACE included serum creatinine level and triple vessel disease.

**Conclusion:**

RA in patients with AMI is feasible with a high procedural completion rate and acceptable periprocedural complications. Given unstable hemodynamics and complex coronary anatomy, the in-hospital and 1-year MACE rates remained higher in patients with AMI compared with patients without AMI.

## Introduction

Rotational atherectomy (RA) is an indispensable tool used for calcified lesion preparation in percutaneous coronary intervention (PCI) (1, 2). In the era of bare-metal stents, RA was once used for aggressive plaque debulking. In the era of drug-eluting stents (DES), stent underexpansion has been shown to associate with worse clinical outcomes and higher risks of stent failure at follow-up (3). The purpose of RA has paradigm-shifted from the merely successful delivery of the stent to adequate modification of plaque, leading to better stent expansion with large minimal stent area (4, 5). RA is widely adopted nowadays for optimal lesion preparation in diverse clinical scenarios, including undilatable or uncrossable lesions (6, 7), non-protected left main lesions (8, 9), side-branch lesions (10), chronic total occlusions (11), complex and high-risk coronary procedures (12), and even in PCI under mechanical circulatory support (13).

In the setting of acute myocardial infarction (AMI), RA has been underused due to several reasons. First, the main mechanism of AMI is plaque rupture with thrombus formation and possible coronary vasospasm. RA was not recommended for treatment with thrombotic lesions (4). Second, RA generates more platelet activation and aggregation, resulting in high-platelet reactivity, which is undesirable in AMI with a prothrombotic state (14, 15). Lastly, the incidence of slow flow or no-reflow phenomenon is higher in RA (4, 16) and could lead to hemodynamic instability or collapse in patients with AMI who already have poor or unstable epicardial coronary flows before RA.

In this study, we aim to evaluate the success rate of RA among patients with AMI, as well as periprocedural complications and major adverse cardiovascular events in a tertiary center.

## Methods

### Patient Population

From January 2009 to March 2018, we enrolled consecutive patients undergoing PCI with RA in our Taichung Veterans General Hospital, a tertiary medical center in Taiwan. Their data were analyzed retrospectively. Patients who met the criteria of current universal definitions of myocardial infarction at the time of PCI (17, 18) were allocated to the AMI group.

Two researchers independently reviewed the computerized electronic medical chart records. Clinical characteristics and biochemical results at the time of hospitalization and during follow-ups were retrieved and recorded in a standardized case record form. Patients who had missed clinical follow-up for more than 3 months were arranged with telephone interviews. For those who died during the study period, we recorded their etiology of death from their death certificates.

The study design and protocol were approved by the Institutional Review Board for Human Research of our institute.

### Angiographic Characterization and Measurements

All angiographies were retrieved from the database in our institute. The lesion characteristics were analyzed using the Rubo DICOM Viewer (version 2.0, build 170828, Rubo Medical Imaging, Aerdenhout, The Netherlands), and the Synergy between PCI with TAXUS and Cardiac Surgery (SYNTAX) scores were calculated for each lesion with at least 50% stenosis of lumen diameter in vessels ≥1.5 mm by an official online calculator at the website. In our study, any significant stenosis of at least 70% stenosis in luminal diameter at non-left main major coronary arteries and at least 50% stenosis at left main coronary artery was defined as coronary artery disease (CAD) and indicated for revascularization anatomically. The other indications of PCI, such as severe ischemia at myocardial perfusion imaging, positive physiological evaluation with fractional flow reserve, or instantaneous wave-free ratio, were at the discretion of interventional cardiologists. In the setting of AMI, the culprit lesions were ascertained by surface ECG, echocardiography, or left ventricular angiogram. Lesions identified by angiography and intracoronary imaging with features suggestive of plaque rupture, plaque erosion, or calcium nodule with or without epicardial coronary flow limitation were also considered as culprit lesions warranting revascularization.

### Procedure Details for RA

Only qualified interventional cardiologists performed RA in our institute. Details of the procedure were reported earlier (10, 13) and were in line with the latest expert consensus regarding RA (4, 5). All patients were pretreated with a standard dose of dual antiplatelet therapy before PCI, as well as calcium channel blocker and nitrate to prevent coronary artery spasm. Indications for RA were either primary (for heavy and circular/rotating intimal calcification or severe fibrotic lesions) or secondary as bailout method (for undilatable or uncrossable lesions).

Rotational atherectomy was executed using Rotablator RA system (Boston Scientific, Marlborough, MA, USA). A 0.014-inch workhorse wire was advanced to the lesion and then exchanged to floppy or extra support RotaWire via a microcatheter. In some lesions uncrossable by microcatheter, bare wiring technique with RoraWire was applied gently and meticulously. A flushing cocktail comprising normal saline, heparin, and isosorbide dinitrate was continuously infused during RA and another bolus of 1,200–1,600 μg of isosorbide dinitrate was given intracoronarily before the activation of RA and stepped burr strategy beginning with an initial 1.25 or 1.5 mm burr at a rotational speed of 170,000–180,000 rpm in most cases. In selective lesions in which burr could not cross easily, a higher speed up to 200,000 rpm was applied. The maximal burr size was determined by the vessel diameter and the effect of adequate debulking, based on either the angiography or intracoronary imaging. After plaque modification by RA, the RotaWire was replaced by a workhorse wire using the same wire-exchange technique. The procedure proceeded with balloon angioplasty with or without stent implantation to achieve optimal angiographic results with minimal residual stenosis. Completion of RA was defined as full debulking of the target lesion without premature termination of RA before proceeding to subsequent treatment.

After stent implantation, dual antiplatelet therapy with aspirin (100 mg/day) and one P2Y12 inhibitor, namely clopidogrel, ticagrelor or prasugrel, were continued for at least 12 months after DES implantation in patients with AMI. In the non-AMI subgroup, the default 6-month duration of DAPT was further adjusted during the follow-up period after weighing the ischemic and bleeding risks.

### Clinical Outcomes

The major adverse cardiac events (MACE) at follow-ups were defined as all-cause death, stroke, non-fatal myocardial infarction, or target vessel revascularization; the cardiovascular major adverse cardiac events (CV MACE) were defined as cardiovascular death, stroke, non-fatal myocardial infarction, or target vessel revascularization. Regular follow-up with invasive angiography was only encouraged and applied to those patients with high anatomical and clinical risks of target vessel failure. Hence, most events of target lesion revascularization in this study were clinically driven.

### Statistical Analysis

Data of continuous variables are expressed as mean ± standard deviation. Categorical variables are expressed as numbers and frequency. Intergroup differences in continuous variables were assessed with unpaired Student's *t*-test, and differences in categorical variables with chi-square test or Fisher's exact tests. Multivariable Cox regression analysis was performed to identify any independent predictors for in-hospital and 1-year CV MACE, respectively. Variables with *p*-values < 0.10 in univariable analysis were assessed using the multivariable model. All statistical analyses were performed using the IBM SPSS statistical packages software for Windows, version 26.0.0.0 (IBM Corp., New York, USA). Two-tailed *p*-values < 0.05 were considered statistically significant.

## Results

### Baseline Characteristics of Patients

During the study period, a total of 411 consecutive patients treated with RA were enrolled in this study (Table 1). In the AMI group, 81% of patients had NSTEMI and 14% presented with cardiogenic shock. Compared with the non-AMI group, patients with AMI were significantly older (76.0 ± 10.8 vs. 72.9 ± 11.4, *p* = 0.011) and had lower level of hemoglobin (10.8 ± 2.4 vs. 11.5 ± 2.0, *p* = 0.001) and lower left ventricular ejection fraction (LVEF) (42.0 ± 11.0 vs. 47.5 ± 13.0, *p* < 0.001). Multivessel disease accounted for 88.4% in the AMI group but only 71.4% in the non-AMI group. Most demographic findings, including sex, hypertension, diabetes, peripheral artery disease, and serum creatinine levels, did not differ between these two groups.

**Table 1:** Demographic data of rotational atherectomy in acute myocardial infarction (AMI) vs. non-AMI cases in the study period.

**Variables**	**AMI** ***N*** **= 121**	**Non-AMI** ***N*** **= 290**	***p*** -value^*^
Sex (M/F)	77/44	192/98	0.617
Age (years)	76.0 ± 10.8	72.9 ± 11.4	**0.011**
Clinical diagnosis (*N*, %)			**<0.001**
Stable angina	0	83 (28.6%)	
Unstable angina	0	147 (50.7%)	
NSTEMI	90 (74.4%)	0	
STEMI	14 (11.6%)	0	
Ischemic CM	0	56 (19.3%)	
Unstable angina + shock	0	2 (0.7%)	
NSTEMI + shock	8 (6.6%)	0	
STEMI + shock	9 (7.4%)	0	
Ischemic CM + shock	0	2 (0.7%)	
Hypertension (*N*, %)	219 (75.5%)	83 (68.6%)	0.147
Diabetes (*N*, %)	77 (63.6%)	164 (56.6%)	0.184
PAD (*N*, %)	10 (8.3%)	34 (11.7%)	0.301
LVEF (%)	42.0 ± 11.0	47.5 ± 13.0	**<0.001**
Lab data			
Hemoglobin (g/dl)	10.8 ± 2.4	11.5 ± 2.0	**0.001**
BUN (mg/dl)	53.2 ± 113.1	31.8 ± 21.8	0.060
Cr (mg/dl)	2.9 ± 2.7	2.7 ± 3.0	0.488
Cholesterol (mg/dl)	145.4 ± 30.2	149.8 ± 32.9	0.266
HDL-chol (mg/dl)	42.9 ± 15.1	45.3 ± 13.1	0.185
LDL-chol (mg/dl)	83.4 ± 26.4	85.8 ± 29.1	0.524
HbA1c (mg/dl)	6.9 ± 1.8	6.6 ± 1.2	0.166
Total CK (U/L)	339.4 ± 507.1	129.9 ± 143.3	**<0.001**
CK-MB (U/L)	16.3 ± 18.6	8.8 ± 9.1	**<0.001**
Troponin (ng/ml)	8.7 ± 15.9	1.2 ± 4.2	**<0.001**
CAD vessels			**0.021**
SVD (*N*, %)	14 (11.6%)	83 (28.6%)	
DVD (*N*, %)	35 (28.9%)	91 (31.4%)	
TVD (*N*, %)	72 (59.5%)	116 (40.0%)	
Plus LM (*N*, %)	19 (15.7%)	37 (12.8%)	
Prior CABG (*N*, %)	8 (6.6%)	12 (4.1 %)	

* *RA in AMI vs. RA in non-AMI*.

*CM, ischemic cardiomyopathy; DVD, double vessel disease; FBS, fasting blood sugar; LVEF, left ventricular ejection fraction; LM, left main coronary artery; PAD, peripheral artery disease; SVD, single vessel disease; TVD, triple vessel disease. Bold values meant Statistically significant (p < 0.05)*.

### Percutaneous Coronary Intervention Procedural Characteristics

Rotational atherectomy was completed in 98.6% of patients with AMI and 98.3% of patients without AMI (*p* = 1.00; Table 2). In both groups, most patients underwent rotablation *via* femoral approach using 7 Fr. sheath, 1.5 mm burr, and were treated with DES. Both groups had similar percentages of heavy calcification, tortuosity, ostial and bifurcation lesions, chronic total occlusion lesions, and ACC/AHA B2/C lesions, whereas stent size was smaller (2.8 ± 0.3 vs. 3.1 ± 2.4, *p* = 0.017) and total lesion length was longer (49.3 ± 25.7 vs. 43.0 ± 23.9, *p* = 0.019) in patients with AMI.

**Table 2:** Demographic and PCI findings of rotational atherectomy in acute myocardial infarction (AMI) vs. non-AMI cases in the study period.

**Variables**	**AMI** ***N*** **= 121**	**Non-AMI** ***N*** **= 290**	***p-*** value^*^
Rotablation vessels			0.237
LM (*N*, %)	1 (0.8%)	0	
LAD (*N*, %)	70 (57.9%)	155 (19.0%)	
LCX (*N*, %)	9 (7.4%)	27 (9.3%)	
RCA (*N*, %)	18 (14.9%)	59 (20.3%)	
LM + LAD (*N*, %)	3 (2.5%)	15 (5.2%)	
LM + LCX (*N*, %)	3 (2.5%)	5 (1.7%)	
LM + RCA (*N*, %)	0	1 (0.3%)	
LAD + LCX (*N*, %)	10 (8.3%)	13 (4.5%)	
LAD + RCA (*N*, %)	2 (1.7%)	11 (3.8%)	
LCX + RCA (*N*, %)	1 (0.8%)	0	
LM + LAD + LCX (*N*, %)	2 (1.7%)	2 (0.7%)	
LM + LAD + RCA (*N*, %)	2 (1.7%)	2 (0.7%)	
Access site			0.060
Radial (*N*, %)	28 (23.1%)	95 (32.8%)	
Femoral (*N*, %)	91 (75.2%)	184 (63.4%)	
Brachial (*N*, %)	2 (1.7%)	11 (3.8%)	
Guide size			0.624
6F (*N*, %)	40 (33.1%)	89 (30.7%)	
7F (*N*, %)	80 (66.1%)	195 (67.2%)	
8F (*N*, %)	1 (0.8%)	6 (2.1%)	
SYNTAX score^#^	35.3 ± 14.0	29.1 ± 14.1	**<0.001**
SYNTAX score post-PCI^#^	11.1 ± 11.3	7.5 ± 9.8	**0.001**
SYNTAX score gain^#^	24.2 ± 11.9	21.6 ± 11.2	**0.036**
Rotablation completed	119 (98.6%)	286 (98.3%)	1.000
Largest burr size			0.403
1.25 mm (*N*, %)	25 (20.7%)	46 (15.9%)	
1.5 mm (*N*, %)	73 (60.4%)	166 (57.2%)	
1.75 mm (*N*, %)	21 (17.4%)	73 (25.2%)	
2.0 mm (*N*, %)	2 (1.7%)	4 (1.4%)	
2.25 mm (*N*, %)	0	1 (0.3%)	
Stenting (*N*, %)	110 (90.9%)	262 (90.3%)	0.862
BMS (*N*, %)	35 (31.8%)	55 (21.0%)	0.131
DES (*N*, %)	75 (68.2%)	205 (78.2%)	
BVS (*N*, %) ?	0	1 (0.4%)	
BMS + DES (*N*, %)	0	1 (0.4%)	
Stent number	2.0 ± 0.9	1.9 ± 0.9	0.339
Stent size (mm)	2.8 ± 0.3	3.1 ± 2.4	**0.017**
Total stent length (mm)	55.6 ± 28.3	51.2 ± 26.8	0.161
Rotablation vessel characteristics			
Total lesion length (mm)	49.3 ± 25.7	43.0 ± 23.9	**0.019**
Heavy calcification	117 (98.3%)	281 (96.9%)	0.521
Tortuosity (*N*, %)	54 (44.6%)	143 (49.3%)	0.386
Ostial lesion (*N*, %)	48 (39.7%)	101 (34.8%)	0.351
Bifurcation (*N*, %)	37 (30.6%)	97 (33.5%)	0.571
Chronic total occlusion	18 (14.9%)	37 (12.8%)	0.566
ACC/AHA lesion B2/C	121 (100%)	286 (98.6%)	0.325
Total contrast dose (ml)	196.8 ± 83.8	194.1 ± 66.6	0.759
Hemodynamic support	35 (28.9%)	38 (9.7%)	**<0.001**

* *RA in AMI vs. RA in non-AMI*.

# *Residual SYNTAX score in patients with prior CABG*.

*BMS, bare metal stent; BVS, bioresorbable vascular scaffold; DES, drug-eluting stent; LAD, left anterior descending artery; LCX, left circumflex artery; LM, left main coronary artery; RCA, right coronary artery. Bold values meant Statistically significant (p < 0.05)*.

The baseline (35.3 ± 14.0 vs. 29.1 ± 14.1, *p* < 0.001), post-PCI (11.1 ± 11.3 vs. 7.5 ± 9.8, *p* = 0.001), and net gain (24.2 ± 11.9 vs. 21.6 ± 11.2, *p* = 0.036) of SYNTAX scores were higher in the AMI group compared with the non-AMI group, implicating more complex coronary anatomy in the AMI group. In addition, the use of hemodynamic support was more frequent in the AMI group (28.9% vs. 9.7%, *p* < 0.001).

A total of 411 patients were selected, of which 405 underwent successful RA. Among them, 372 patients were treated with stenting after rotablation (91.9%) and 33 were left unstented. The reasons why we did not perform stenting were rotablation for side branches (12 patients, 36.4%), diffuse and small lesions without adequate stent landing zone (7 patients, 27.3%), and in-stent restenosis (5 patients, 15.2%; most of them were treated with drug-eluting balloon), chronic total occlusions with negative vessel remodeling in the distal vessel that was too small to be stented with confidence (5 patients, 15.2%), and patient factors (2 patients, 6.1%; one was supposed to undergo urgent non-cardiac surgery right after PCI, another patient could not cooperate with the procedure after successful rotablation and plain old balloon angioplasty (POBA)), operator discretion (2 patients, 6.1%; one patient had slow-flow phenomenon after rotablation and POBA, another one was treated with cutting balloon at the discretion of the operator).

### Procedure Outcomes

Overall, no difference was observed in the incidence of acute no-flow phenomenon, vessel perforation, wire fracture, and profound in-procedure shock between the AMI and non-AMI groups (Table 3). No patient died or needed emergent CABG during the procedure. Nevertheless, the AMI group had a higher incidence of ventricular arrhythmia (5.8% vs. 0.7%, *p* = 0.003).

**Table 3:** Procedure outcomes of rotational atherectomy in acute myocardial infarction (AMI) vs. non-AMI cases in the study period.

**Variables**	**AMI** ***N*** **= 121**	**Non-AMI** ***N*** **= 290**	***p-*** value^*^
Acute no flow (*N*, %)	10 (8.3%)	25 (8.6%)	0.906
Perforation (*N*, %)	2 (1.7%)	3 (1.0%)	0.634
Wire transection (*N*, %)	1 (0.8%)	0	N/A
Profound/ refractory shock	19 (15.7%)	31 (10.7%)	0.156
Ventricular arrhythmia (*N*, %)	7 (5.8%)	2 (0.7%)	**0.003**
Emergent CABG (*N*, %)	0	0	N/A
Die on table (*N*, %)	0	0	N/A

* *RA in AMI vs. RA in non-AMI*.

*CABG, coronary artery bypass graft. Bold values meant Statistically significant (p < 0.05)*.

### In-hospital and Clinical Outcomes up to 1 Year

The in-hospital and clinical outcomes at different time points are presented in Table 4. For all patients who underwent RA in the setting of AMI, the in-hospital, 30-day, 90-day, and 1-year CV MACE rates were significantly higher than those in the non-AMI group (in-hospital 13.2 vs. 2.8%, *p* < 0.001; 30-day 14.2 vs. 4.5%, *p* < 0.001; 90-day 20.8 % vs. 6.9%, *p* < 0.001; 1-year 30.8%, 19.1%, *p* = 0.01). Patients in the AMI group also had significantly higher MACE, death, and CV death up to 1 year. No difference was found between the two groups regarding in non-fatal myocardial infarction, target vessel revascularization, stroke, or stent thrombosis rates.

**Table 4:** Clinical outcomes of rotational atherectomy in acute myocardial infarction (AMI) vs. non-AMI cases in the study period.

**Variables**	**AMI** ***N*** **= 121**	**Non-AMI** ***N*** **= 290**	***p-*** value^*^
In-hospital			
MACE^#^ (*N*, %)	22 (18.2%)	9 (3.1%)	**<0.001**
CV MACE^†^ (*N*, %)	16 (13.2%)	6 (2.8%)	**<0.001**
Death (*N*, %)	21 (17.4%)	6 (2.1%)	**<0.001**
CV death (*N*, %)	14 (11.6%)	3 (1.0%)	**<0.001**
Non-fatal MI (*N*, %)	2 (1.7%)	1 (0.3%)	0.208
Stent thrombosis	1 (0.8%)	0	0.294
Stroke (*N*, %)	0	1 (0.3%)	1.000
TLR (*N*, %)	0	0	N/A
TVR (*N*, %)	1 (0.8%)	2 (0.7%)	1.000
30-day			
MACE (*N*, %)	25 (20.8%)	16 (5.5%)	**<0.001**
CV MACE (*N*, %)	17 (14.2%)	13 (4.5%)	**0.001**
Death (*N*, %)	23 (19.2%)	10 (3.4%)	**<0.001**
CV death (*N*, %)	14 (11.7%)	7 (2.4%)	**<0.001**
Non-fatal MI (*N*, %)	2 (1.7%)	1 (0.3%)	0.206
stent thrombosis	2 (1.7%)	1 (0.3%)	0.206
Stroke (*N*, %)	0	2 (0.7%)	1.000
TLR (*N*, %)	1 (0.8%)	2 (0.7%)	1.000
TVR (*N*, %)	2 (1.7%)	4 (1.4 %)	1.000
90-day			
MACE (*N*, %)^§^	36 (30.0%)	27 (9.3%)	**<0.001**
CV MACE	25 (20.8%)	20 (6.9%)	**<0.001**
Death (*N*, %)	27 (22.5%)	15 (5.2%)	**<0.001**
CV death (*N*, %)	15 (12.5%)	8 (2.8%)	**<0.001**
Nonfatal MI (*N*, %)	4 (3.3%)	2 (0.7%)	0.064
Stent thrombosis	2 (1.7%)	1 (0.3%)	0.207
Stroke (*N*, %)	0	2 (0.7%)	1.000
TLR (*N*, %)	6 (5.0%)	7 (2.4%)	0.215
TVR (*N*, %)	8 (6.7%)	10 (3.5 %)	0.150
1-year			
MACE (*N*, %)^§^	57 (47.5%)	74 (25.7%)	**<0.001**
CV MACE	37 (30.8%)	55 (19.1%)	**0.01**
Death (*N*, %)	41 (34.2%)	42 (14.5%)	**<0.001**
CV death (*N*, %)	17 (14.2%)	16 (5.5%)	**0.004**
Nonfatal MI (*N*, %)	6 (5.0%)	7 (2.4%)	0.215
Stent thrombosis	3 (2.5%)	1 (0.3%)	0.078
Stroke (*N*, %)	0	4 (1.4%)	0.326
TLR (*N*, %)	16 (13.3%)	32 (11.1%)	0.518
TVR (*N*, %)	20 (16.7%)	37 (12.8%)	0.304

* *RA in AMI vs. RA in non-AMI*.

# *Death, nonfatal myocardial infarction, stroke, or target vessel revascularization (TVR)*.

† *Cardiovascular death, nonfatal myocardial infarction, stroke, or target vessel revascularization (TVR)*.

*One patient in RA in the AMI group was lost to follow-up after discharge from ward*.

§ *Another patient in RA in the non-AMI group was lost to follow-up after 1 month*.

*MACE, major adverse cardiovascular events; TLR, target lesion revascularization; TVR, target vessel revascularization. Bold values meant Statistically significant (p < 0.05)*.

### Multivariable Logistic Regression Analysis for In-hospital and 1-Year CV MACE

The multivariable analysis identified independent predictors for in-hospital CV MACE as follows: age, female sex, peripheral artery disease, AMI at presentation, cardiogenic shock, and post-PCI SYNTAX score (Table 5). In the multivariable analysis for 1-year CV MACE, AMI at presentation [odds ratio (OR) 1.79; 95% CI 1.02–3.15; *p* = 0.042) and cardiogenic shock (OR 2.41; 95% CI 1.29–4.53; *p* = 0.006) remained as independent predictors. Serum creatinine level (OR 1.12; 95% CI1.03–1.22; *p* = 0.009) and triple vessel disease (compared to single-vessel disease; OR 2.75; 95% CI 1.16–6.52; *p* = 0.022) were the other predictors for 1-year CV MACE (Table 5).

**Table 5:** Predictors of cardiovascular major adverse cardiovascular events (CV MACE) during hospitalization and at 1-year follow-up from the multivariable models.

**Predictors**	**In-hospital CV MACE**			**1-year CV MACE**		
	**Adjusted OR**	**95% CI**	***p-*** **Value**	**Adjusted OR**	**95% CI**	***p-*** **value**
**Age**	1.10	1.02–1.18	**0.017**	0.99	0.97–1.02	0.590
**Male gender**	0.23	0.07–0.75	**0.015**	0.70	0.42–1.18	0.184
**Hypertension**	1.42	0.42–4.79	0.577	0.89	0.51–1.55	0.687
**Diabetes**	0.79	0.26–2.45	0.688	1.11	0.66–1.87	0.687
**PAD**	1.06	1.54–23.18	**0.010**	1.09	0.49–2.42	0.831
**Serum creatinine**	1.06	0.85–1.32	0.627	1.12	1.03–1.22	**0.009**
**AMI**	12.72	2.86–56.58	**0.001**	1.79	1.02–3.15	**0.042**
**Cardiogenic shock**	9.18	2.15–39.24	**0.003**	2.41	1.29–4.53	**0.006**
**SVD**	Reference			Reference		
**DVD**			0.996	1.77	0.76–4.13	0.186
**TVD**			0.996	2.75	1.16–6.52	**0.022**
**Hemodynamic support**	1.87	0.48–7.25	0.364	1.42	0.71–2.83	0.322
**SYNTAX score**	0.98	0.92–1.04	0.497	0.99	0.97–1.02	0.410
**SYNTAX score post-PCI**	1.07	1.01–1.13	**0.022**	1.01	0.98–1.04	0.669

*95% CI, 95% confidence interval; ACS, acute coronary syndrome; CM, cardiomyopathy; DM, diabetes mellitus; DVD, double vessel disease; OR, odds ratio; PAD, peripheral artery disease; SVD, single vessel disease; disease; TVD, triple vessel disease. Bold values meant Statistically significant (p < 0.05)*.

## Discussion

In summary, our retrospective study revealed several important findings regarding RA among patients with AMI in the modern era: (1) RA in the setting of AMI is safe and feasible, associated with high procedural success and acceptable periprocedural complications; (2) the in-hospital, 30-day, 90-day, and 1-year CV MACE rates in the AMI group were significantly higher than non-AMI group; (3) AMI at initial presentation, cardiogenic shock, age, female sex, peripheral artery disease, and post-PCI SYNTAX score were independent predictors for in-hospital CV MACE; whereas, AMI at initial presentation and cardiogenic shock remained as predictors of 1-year CV MACE, as well as serum creatinine level and triple vessel disease.

According to a national cohort study on US Veterans, the proportion of patients undergoing PCI for calcification lesions has been on the rise recently (19). Patients with severe calcification had significantly more major adverse cardiac events after PCI compared with those without (20). Hence, how to deal with a calcified plaque by different tools to get good lesion preparation in PCI has attracted more attention lately (21, 22). Clinical use of RA for heavy calcified or severe fibrotic lesions accounted for 0.8–3.1% among patients undergoing PCI in European countries (5). Among patients undergoing RA, the percentage of acute coronary syndrome (ACS) ranged from 20 to 37% in several studies focused on RA in ACS (23–26). In this study, we enrolled patients with AMI with a stricter definition and only patients with elevated high-sensitivity cardiac troponins were included. Patients with AMI were near 30% of patients undergoing RA in our cohort, a proportion that is comparable with previous studies (23–26). The previous studies reported that RA in patients with ACS had a high procedure completion rate, comparable with patients without ACS. To our knowledge, our cohort had the largest number of patients with AMI undergoing RA in a single center. Patients in our cohort also carried more high-risk clinical features with a mean age of 76 years old, 63.6% with diabetes, as well as more high-risk anatomical features with 88.4% multivessel disease and extremely high syntax score with a mean of 35.3 compared with previous studies. Nevertheless, our results still demonstrated a similar procedure success rate, reassuring the feasibility of RA in these high-risk patients.

In a single-center cohort in Germany, including 8 STEMI and 100 NSTE-ACS patients treated with RA, the 2-year MACE rate was higher in patients with ACS compared with 433 patients with stable CAD (39.9 vs. 22.4%, log-rank *p* = 0.002; hazard ratio (HR) 1.39; 95% CI: 1.12–1.73; *p* = 0.003) (23). In our study, despite the comparable procedural success rates of RA in the AMI and non-AMI groups, we still found higher in-hospital and 1-year CV MACE rates in the AMI group. The poor outcome in the AMI group could be attributed to unstable hemodynamic and vulnerable plaques in the setting of AMI, as well as high clinical and anatomical risks in the AMI group. In our study, patients in the AMI group were older and had smaller stent size, longer total lesion length, higher baseline and residual SYNTAX scores, as well as more frequent use of hemodynamic support compared with the non-AMI group. All the above characteristics were known unfavorable factors for MACE after PCI. From an analysis in patients with ACS undergoing RA derived from the ROTational AThErectomy (ROTATE) registry, MACE after a median of 27.9 months was significantly higher in the NSTE-ACS group compared with the stable angina group (32.4 vs. 24.2%, log-rank *p* < 0.001), but this difference no longer persisted after propensity score matching (25), implicating that higher risk profiles other than ACS *per se* in the NSTE-ACS group contributed to the poor clinical outcomes.

Recently, a prospective European multicentral registry (Euro4C registry) demonstrated a high clinical success in 91.9% of rotablation. Factors independently associated with 1-year MACE were female gender, renal failure, ACS at admission, depressed LVEF, and left main lesion (26). In our study, the AMI at initial presentation and serum creatinine level were found to be independent predictors for 1-year CV MACE, in line with the recent Euro4C registry. Of note, the Euro4C registry indicated that women had worse clinical outcomes following RA during hospitalization and at 1-year follow-up. However, the procedural complications did not significantly differ between genders, and the reasons for poor clinical outcomes in women following RA remained unknown (27). In our study, female gender was a predictor for in-hospital CV MACE but not for 1-year CV MACE. Further studies focusing on gender difference of patients undergoing RA are warranted to clarify the relationship of gender and clinical outcomes of RA.

Of interest, 98.3% of patients had heavily calcified lesions and 44.6% had torturous lesions in our AMI group. Nevertheless, the perforation rate of RA was only 1.7%, comparable with other RA studies (4). In addition to meticulous skills and experienced hands, another crucial point is that we learned from mistakes. The mechanism of perforation was sought and discussed case by case in a formal conference in our institute (28). Knowing why perforation occurs in RA could help operators avert such disasters and maintain lower complication rates.

On the other hand, the incidence of slow flow or no-reflow phenomenon in our AMI group was 8.3%, higher than the German cohort with an event rate of 0.8% (23) or ROTATE registry in the setting of ACS with an event rate of 3.3% (25). The difference was probably attributed to the definition among these studies. In the German cohort, only persistent slow flow or reflow at the end of the procedure was documented (23), whereas in our study, any transient slow flow or no-reflow during the procedure was counted when we retrospectively reviewed the angiography in detail. Nevertheless, most slow flow or no-reflow events in our cohort were relieved by intracoronary use of adenosine without persistent hemodynamic deterioration. The risk of slow flow or no-reflow was also comparable between our AMI and non-AMI groups, supporting that RA is a relatively safe procedure in AMI.

### Study Limitations

Our study had several limitations. First, the retrospective design was inherently associated with selection bias and other confounding factors. Some critical parameters, such as LVEF and detailed analysis of intracoronary imaging, could not be collected well in every patient and utilized for outcome analysis. Second, the enrollment of consecutive all-comers, especially those with unstable hemodynamics at initial presentation, might influence the clinical results. However, this allowed us to investigate the safety and efficacy of RA in AMI in real-world practice and confirmed the feasibility in this complex scenario. Third, the incidence of RA-associated periprocedural myocardial infarction in our patients was difficult to determine, given that we only recruited patients with AMI with positive troponin assays. Despite cardiac enzymes being regularly followed up after RA in our cohort, we could not differentiate the extent of myocardial injury from AMI *per se* or from the procedure of rotablation. Fourth, although our study enrolled 23 patients presenting with STEMI and was probably the largest cohort in single center to date for this unique group (12, 23, 24, 26, 29), the enrolled number was still limited and the amount of thrombus burden could not be precisely measured. The application of RA in moderate to large burden of thrombus remains to be confirmed in larger studies for STEMI.

## Conclusion

Despite very high-risk clinical and anatomical features in patients with AMI, RA was feasible with comparable high procedure success and low complications compared with the patients without AMI. The incidence of in-hospital and 1-year CV MACE events was still higher in the AMI group compared with the non-AMI group. AMI at initial presentation and cardiogenic shock were predictors of both in-hospital CV MACE and 1-year CV MACE for those undergoing RA in the study periods.

## Data Availability Statement

The raw data supporting the conclusions of this article will be made available by the authors, without undue reservation.

## Ethics Statement

The studies involving human participants were reviewed and approved by Institutional Review Board for Human Research of Taichung Veterans General Hospital. The patients/participants provided their written informed consent to participate in this study.

## Author Contributions

W-LL and Y-WC contributed to the conception and design of the study. C-HL, C-SS, W-CC, C-YW, W-JC, T-HL, K-WL, and T-JL contributed to data collection. W-LL analyzed and interpreted the data. Y-WC drafted the report, which was critically revised for important intellectual content by W-LL. All authors have participated in the work, have reviewed and agreed with the content of the article, and approved the final version of the report.

## Conflict of Interest

The authors declare that the research was conducted in the absence of any commercial or financial relationships that could be construed as a potential conflict of interest.

## Publisher's Note

All claims expressed in this article are solely those of the authors and do not necessarily represent those of their affiliated organizations, or those of the publisher, the editors and the reviewers. Any product that may be evaluated in this article, or claim that may be made by its manufacturer, is not guaranteed or endorsed by the publisher.
